# The Role of Postoperative Radiation and Chemoradiation in Merkel Cell Carcinoma: A Systematic Review of the Literature

**DOI:** 10.3389/fonc.2013.00276

**Published:** 2013-11-14

**Authors:** Shaakir Hasan, Liyu Liu, Jacob Triplet, Zhen Li, David Mansur

**Affiliations:** ^1^College of Osteopathic Medicine, Nova Southeastern University, Fort Lauderdale, FL, USA; ^2^Department of Neuroscience, Yale University, Hartford, CT, USA; ^3^Department of Radiation Oncology, University Hospitals at Case Western, Cleveland, OH, USA

**Keywords:** Merkel cell carcinoma, adjuvant radiotherapy, chemoradiation, postoperative radiation Merkel, review

## Abstract

**Objective:** A systematic review of the literature was undertaken to investigate whether adjuvant radiotherapy and/or chemotherapeutics offered any additional benefit than surgery alone in the treatment of Merkel Cell Carcinoma (MCC).

**Methods:** A PubMed, MEDLINE search was conducted between 1995 and 2013, to identify reported cases of surgically treated MCC followed by either observation, radiation, or chemoradiation. Patient demographics and outcomes were recorded and compared in a systematic fashion.

**Results:** Thirty-four studies (*n* = 4475) were included. The median age was 73 years, median follow up was 36 months and there was a 1.5:1 ratio of men to women. All 4475 patients had surgery, 1975 had no further treatment, 1689 received postoperative RT, and 301 received postoperative chemoRT. The most common site was face/head/neck, 47.8%. Stage 1 was the most common clinical stage at diagnosis (57%). Three-year local control was 20% (median 10%) in the observation cohort, compared to 65% (62%) with postoperative RT, and 67% (75%) with postoperative chemoRT; these findings were statistically significant (*P* < 0.001). Recurrence was found to be 38% (60%) in the observation cohort, compared to 23% (20%) with postoperative RT (*P* < 0.001). Three-year overall survival (OS) was found to be 56% (57%) in the observation cohort, compared to 70% (78%) with postoperative RT and 73% (76%) with postoperative chemoRT (*P* < 0.001). The observation cohort had a median OS of 44 months compared with 64 months (*P* < 0.001) in the postoperative RT cohort. There was no statistically significant difference in any parameters assessed between postoperative radiation and postoperative chemoradiation arms.

**Conclusion:** The comprehensive collection of retrospective data suggests a survival and control benefit for postoperative radiation in MCC. No differences were noted between adjuvant radiation and chemoradiation. This analysis indicates the need for prospective trials with patients stratified by known prognostic factors.

## Introduction

Merkel cell carcinoma (MCC) is an aggressive cutaneous malignancy that is known for its ability to metastasize, its high recurrence rate, and a mortality rate greater than that of melanoma. Merkel cells, first described in 1875 by Friedrich Merkel, are believed to be mechanoreceptors that relay information regarding light touch and hair movement (1, 2) Controversy exists as to the origin of these mechanoreceptors; both neural crest and epithelial origin have been suggested (3, 20) Regardless of its embryologic origin, its malignant transformation has devastating potential.

Merkel cell carcinoma is relatively rare, with an annual incidence rate of 0.6 per 100,000 (4). It affects nearly twice as many men as women and is more prevalent in whites than blacks, 94 and 1%, respectively (1, 4, 32). The average age of presentation for this malignancy is 72 years (1). The mean age of prevalence decreases dramatically, to 53 years of age, for immunocompromised individuals. Individuals with CLL, HIV/AIDS, and organ transplant recipients are at a 30, 13, and 10-fold increased risk respectively (12, 21, 36).

Merkel cell is prevalent in sun-exposed areas, with nearly half of all incidences occurring in the head and neck region (29). In addition to sun-exposure, MCC has been associated with p-53 mutations, arsenic exposure, Methoxsalen and ultraviolet-A treatment in psoriasis, and infrared skin damage (1, 12, 13, 29, 48). Although these associations have been publicized, MCC has its strongest association with polyomavirus, present in 80% of cases (48).

The National Comprehensive Cancer Network (NCCN) 2013 guidelines recommend that patients with biopsy proven MCC undergo sentinel lymph node biopsy (SLNB) and appropriate immuno panel with wide local excision (WLE) of the primary tumor. The NCCN 2013 guidelines (http://www.nccn.org/professionals/physician_gls/pdf/mcc.pdf) do not provide definitive recommendations for treatment of the various clinical stages of MCC. However, treatment options are still often based on the clinical stage of the cancer and consist of excision, radiation therapy, chemotherapy, or any combination of the three (38, 44). Traditionally, MCC is treated surgically, followed by radiation therapy in some instances although the radiosensitive nature of the tumor is not definitively established (10, 19, 27). Radiation therapy alone may be used for patients who are not surgical candidates (38). The rationale for concomitant postoperative (chemoradiation) is that MCC is known to have chemosensitive based on, high initial response rates in metastatic settings (9, 16). Poulsen et al. (38) however demonstrated no significant difference in survival benefits with adjuvant chemotherapeutics compared with radiation therapy alone (40). Chemotherapy is typically reserved for patients with high risk of distant metastatic disease or those with existing metastatic disease.

Data supports the use of a 1- to 2-cm margin for excision, although this remains controversial (5, 6, 8, 30). Alternative surgical options, such as the Moh’s micrographic surgery, are also available. The Moh’s technique has become increasingly popular due to its preservation of tissue, a cosmetic advantage, and has been shown to be comparable to that of WLE (7). Due to the lack of consensus for the treatment of MCC, a literature search was undertaken to investigate postoperative treatment modalities for MCC.

## Materials and Methods

### Study selection

A PubMed, MEDLINE search was conducted between the years 1995–2013, to identify reported cases of surgically treated MCC. The following keyword combinations were used: MCC, surgery, postoperative management, combination treatment, radiation therapy, and chemoradiation therapy. The search was limited to studies published in English. A title and abstract review was then performed and reports from additional secondary references were also manually reviewed. Studies were included for their relevance to surgery only (observation), surgery combined with postoperative radiotherapy (RT) and/or surgery combined with postoperative chemoradiotherapy (chemoRT). Study types including retrospective, prospective and case series were reviewed. Commentaries, editorials, and review articles were excluded. WLE, defined as removal of the cancer accompanied by surrounding tissue, and Moh’s micrographic surgery, defined as progressive removal of cancer-containing skin layers until only cancer-free tissue remains, were the majority of the surgical techniques included in this review. In cases where radiotherapy was used, radiation was localized and the dose was between 45 and 65 Gy (median of 50 Gy), with 3–4 cm margins. Chemotherapeutics used in the literature include carboplatin, etoposide, cyclophosphamide, doxorubicin, epirubicin, vincristine, cisplatin, capecitabine, or any combination.

### Data extraction

Patient characteristics including age, gender, size and location of primary tumor, clinical stage, and nodal involvement were reported. The following system was used to standardize clinical stages: stage I indicated localized primary tumor of any size, without evidence of lymph node involvement or distant metastasis; Stage II indicated regional lymph node involvement (regional draining lymph nodes or adjacent lymph nodes) without distant metastasis; Stage III was evidence of distant disease. Primary treatment were carefully reviewed and categorized into three modalities: observation, RT, and chemoRT. Assessment of outcomes were reviewed and reported as overall survival (OS), OS after 1 year, after 3 years, local control (LC) after 1 year, 3 years, crude recurrence (including local recurrence and/or nodal/distant relapse), time to recurrence, and toxicity.

### Inclusion criteria

The following inclusion criteria were applied to each study:

- Primary tumor of MCC in any stage
- Positive or negative metastases to lymph nodes
- Lesions of any size
- Primary treatment included curative surgery (Moh’s micrographic surgery or WLE) followed by observation, surgery followed by radiation within 3 months, or surgery followed by concurrent chemoradiation within 3 months.

### Exclusion criteria

- Unknown primary tumor
- Treatment of recurrent MCC
- Primary treatment did not include surgery
- Radiation or chemoradiotherapy used as salvage therapy
- Insufficient documentation.

### Statistical analysis

Treatment modalities were correlated to different outcome. *T*-tests, odds ratios, and chi square testing was used when appropriate using the software SOFA and MedCalc.

## Results

### Study selection

**Figure 1:**
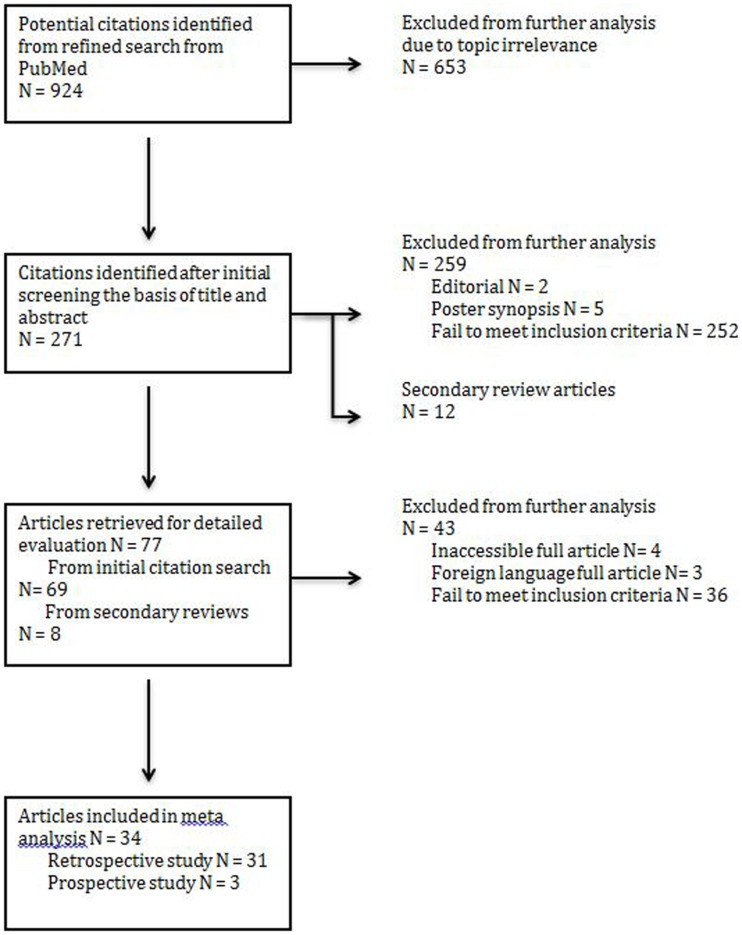
**PRISMA (preferred reporting items for systemic reviews and meta-analysis) flow chart illustrating the identification of articles for analysis**.

An initial search yielded 271 records from PubMed. An additional 12 secondary references were also reviewed (Figure 1). Abstract review and manual review from an additional 12 secondary references were performed and 34 studies (a total patient number of *n* = 4475) were included for their fulfillment of inclusion criteria listed above and relevance to the three treatment modalities of interest (Figure 1). Out of the 34 studies, there were 31 retrospective studies (a total patient number of *n* = 4315); 2 studies reported surgery combined with chemoradiotherapy (*n* = 46), seven studies reported treatment modalities of surgery alone vs. surgery combined with post-op radiotherapy (*n* = 2220), and 22 studies reported all three treatment modalities of interests (*n* = 2049). Out of the 34 studies, there were three prospective studies; two studies evaluated post-op chemoradiotherapy only (*n* = 58) while one study evaluated all three treatment modalities of interest (*n* = 102). Not all cases of MCC treated by surgery could be included in every parameter assessed.

### Demographics

The median age of patients included in analysis was 73 years (63–80) for all three treatment arms and there was a 1.5:1 ratio of men to women. The median follow up period was 36 months (range 7–60). All 4475 patients included in the review had surgery, 1975 patients had no further treatment (observation), 1689 received postoperative radiotherapy (RT), and 301 received postoperative chemoradiotherapy (chemoRT). The most common primary site was the face/head/neck, representing 47.8% (*n* = 1327) of all cases (Table 1). The most common clinical stage at diagnosis was Stage I local disease, which represented 57% (*n* = 2037) of all cases, although this was not equally represented in each cohort. Stage 1 disease represented 67% and 60% of all cases that received observation and radiation therapy post-surgery, respectively, but represented 29% of all cases that received postoperative chemoradiotherapy. Stage 2, which indicates clinical involvement of regional lymph nodes represented 30% overall, 39% of RT cohort and 57% of postoperative chemoRT cohort. Report of nodal status (clinical and histological) and treatment of regional nodes (positive or negative) included regional or radical dissection, biopsy, and irradiation. Most nodal treatments were dependent on clinician preference and was inconsistent across studies.

**Table 1:** **Summary of patient demographics**.

Characteristics	Total/weighted mean	Observation		Surgery + RT		Surgery + ChemoRT	
Patient	4775	1975	%	1689	%	301	%
Gender
Male	2663	589	57	513	59	117	73
Female	1679	451	43	360	41	44	27
Age
Median (years)	73.0	75.1		72.1		66.1	
Follow up
Median (months)	35.8	50.2		47.4		25.8	
Clinical stage
I	2094	720	67	650	60	36	36
II	1006	347	33	423	39	55	54
III	276	3	0.2	2	0.2	10	10
Nodal involvement (clinical)
Positive	715	457	35	323	49	132	67
Negative	940	903	66	333	51	65	33
Location of primary tumor
Extremities	993	148	45	161	41	58	36
Face/head/neck	1327	151	46	177	45	66	41
Trunk	262	31	9	54	14	8	5
Other	197	0	0	0	0	28	18

*Observation, surgery only treatment group; RT, received postoperative radiation therapy; ChemoRT, received postoperative chemoradiation therapy*.

### Local control and crude recurrence

Local control was defined as the percentage of patients with no evidence of disease at the primary site or regional lymph nodes at follow up. The weighted mean for 3-year LC rate was 20% in the observation cohort with a median of 10%. This is compared to a median of 66% of patients achieving LC (*P* < 0.001) after receiving postoperative radiation therapy. Three-year LC rate was 67% with postoperative chemoradiation with a median of 75% in comparison to 20% in the observation cohort (*P* < 0.001). Crude recurrence was defined as any documented recurrence of neoplasm including local, nodal, or distant metastatic recurrences anytime during the follow up period. In the observation group, there was a 38% recurrence rate (with a median of 60%) compared to a 23% recurrence in of the postoperative radiotherapy group (median of 20%) (*P* < 0.001) (Table 2). There was no statistically significant difference in any control or recurrence parameters assessed between postoperative radiation and postoperative chemoradiation arms (Table 3).

**Table 2:** **Outcome summary of retrospective studies**.

	Observation	Median	Surgery + RT	Median	Surgery + ChemoRT	Median
	44		64		84^a^	
1 year Overall survival (%)	81	89	90	90	89	100
3 year Overall survival (%)	56	57	70	78	73	76
Crude recurrence (%)	38	60	23	20	22	35
Time to recurrence (months)	9		16		170	
1 year Local control (%)	41	30	84	91	80	100
3 year Local control (%)	20	10	65	62	69	75

*Observation, surgery only treatment group; RT, received postoperative radiation therapy; ChemoRT, received postoperative chemoradiation therapy*.

*^a^Only one point of reference*.

**Table 3:** **Outcome summary of prospective studies**.

	Observation	Surgery + RT	Surgery + ChemoRT (%)
Acute toxicity	–	–	45
Chronic toxicity	–	–	17^a^
Crude recurrence	–	42%^a^	31

*Observation, surgery only treatment group; RT, received postoperative radiation therapy; ChemoRT, received postoperative chemoradiation therapy*.

*^a^Only one point of reference*.

### Survival

The 1-year OS was 81% with the median of 89% in the observation cohort in comparison to 90% and a median of 90% the postoperative radiation cohort (*P* < 0.001). The 1-year OS in the postoperative chemoradiation therapy cohort was 89% (*P* < 0.001) in comparison to observation with a median of 100%. The 3-year OS was 55% in observation (median of 57%) in comparison to 70% (median of 78%) in the postoperative radiation cohort (*P* < 0.001), and 73% (median of 76%) in postoperative chemoradiation cohort when compared to observation (*P* < 0.001). The median OS in months was 44 months in the observation cohort in comparison to 64 months in the postoperative radiation cohort (*P* < 0.001). Again there were no statistically significant differences in any survival parameters assessed between postoperative radiation and postoperative chemoradiation arms (Table 3).

### Subset analysis

A subset analysis was performed contrasting observation and RT treatments based on the size of the tumor; chemoRT was excluded due to lack of data. Collected data was separated into two groups (either <2 cm or ≧2 cm) based on the mean tumor size for the study; it does not indicate that all tumors in the study were <2 cm or ≧2 cm. Only studies where mean tumor size could be attained were included in this subset analysis.

**Figure 2:**
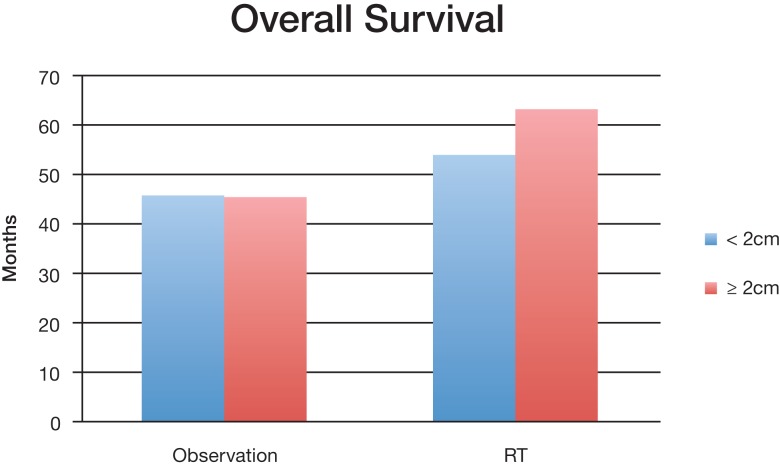
**Median overall survival of different size tumors between observation and postoperative radiation therapy cohort**.

**Figure 3:**
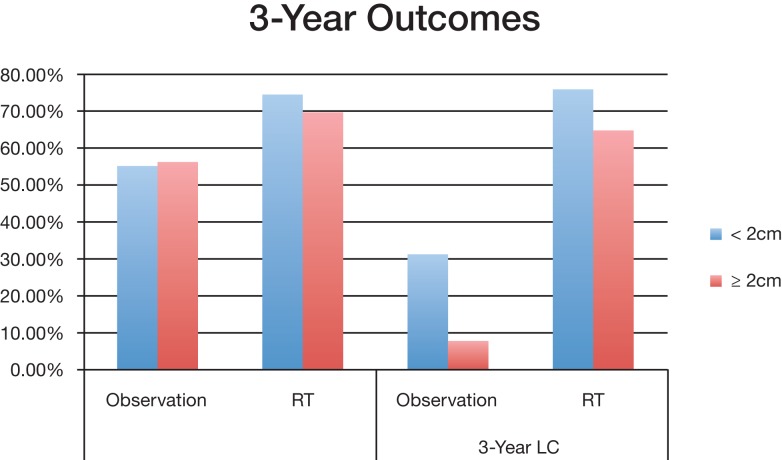
**Overall survival and local control of different tumor sizes between observation and postoperative radiation therapy cohort**. Abbreviations: observation, surgery only treatment group; RT, received postoperative radiation therapy; OS, overall survival; LC, local control.

Group 1 (<2 cm) median overall survival for postoperative observation group and RT group was 45.7 months and 53.9 months (*P* < 0.001) respectively; Group 2 (≧2 cm) OS was 45 months and 63 months (*P* < 0.001) respectively (Figure 2). For tumors <2 cm, the 3-year OS was 55.4% and 74.6% (*P* < 0.001) for observation and postoperative radiation respectively. Similar trends were also noted for tumors >2 cm for 3-year OS (56.4% with observation vs. 69.8% with radiation) (Figure 3). The <2 cm 1-year local control rate (LC) was 52.9% and 89.2% (*P* < 0.001) for observation and radiation, respectively; and the difference was even more pronounced for the tumors >2 cm (38% vs. 83%). Similarly, the 3-year LC for tumors <2 cm was 31.6% and 75.9% (*P* < 0.001) for observation and radiation, respectively; compared to 8% with observation and 64.7% with radiotherapy (*P* < 0.001) for tumors >2 cm (Figure 3).

## Discussion

The diagnosis of MCC, made difficult by its lack of clinical symptoms, has devastating potential. Immunostains have contributed greatly to earlier diagnosis of this rare cancer. Despite the modern advances in diagnostic abilities, there is little uniformity of the treatment protocol for MCC. Surgical excision of the primary tumor is most commonly practiced with little dispute. However, few guidelines exist to direct treatment based on variables such as clinical stage and size of the primary tumor. To our knowledge, no definitive recommendations exist for the adjuvant treatment of radiotherapy and/or chemotherapeutics. MCC is known to be a radiosensitive cancer, though adjuvant radiotherapy is at the discretion of the treating physician. Chemotherapeutics have been proven to have an initial response in metastatic setting (2), but its use in the treatment of localized MCC is controversial.

This systematic review supports the use of adjuvant treatment with surgical excision based on the retrospective data (Table 4). The data suggests a benefit of postoperative radiation for MCC in terms of OS. Adjuvant radiotherapy 3-year OS was 14.2% greater for the radiotherapy arm (70.4%) than the observation group (56.2%). Upon subset analysis, studies with an average tumor <2 cm demonstrated a 3-year OS that was 19.2% greater for the radiotherapy arm (74.6%) than the observation group (55.4%). Studies with tumors ≧2 cm demonstrated a 3-year OS that was 13.4% greater for the radiotherapy arm (69.8%) than the observation group (56.4%).

**Table 4:** **Summary of age, follow up, size, nodal status, and outcome across three treatment cohort from 34 original studies**.

Study	PostOp Arm	F/U	*N*	Age	AvgSize	LN+ (%)	LN−(%)	Crude Rec (%)	3-year LC (%)	3-year OS (%)
Field et al. (15)	Observation	43.2	256	70	1.2	38	62	25	–	–
	Radiation		68					2.7	–	–
	ChemoRT		27					–	–	–
Senchenkov et al. (43)	Observation	4.8	16	66.6	–	–	–	50	–	–
	Radiation		21					38	–	–
Warner et al. (49)	Observation	16	8	74	1.5	27	72	88	–	–
	Radiation		8					33	–	–
Meeuwissen et al. (31)	Observation	20	38	74	<2	–	_	100	0	65
	Radiation		34					29	68	86
Ghadjar et al. (17)	Observation	60	40	73	2.4	–	–	–	8	56
	Radiation		118						62	64
	ChemoRT		15					–	–	–
Howels et al. (22)	Observation	38	53	75	1.1	29	66	–	–	–
	Radiation		69					–	–	–
	ChemoRT		5					–	–	–
Mojika et al. (33)	Observation	40	689	74	2.0	–	–	–	–	55
	Radiation		477					–	–	69
Ott et al. (34)	Observation	37	–	68	2.0	26	74	48	–	–
	Radiation		–					0	–	50
Poulsen et al. (40)	Observation	48	0	67	>1	–	–	–	–	–
	Radiation		0					–	–	–
	ChemoRT		41					48	75	76
Akhtar et al. (2)	Observation	7	5	70.3	<2	–	–	–	–	–
	Radiation		–					–	–	–
Teratola et al. (47)	Observation	39.6	53	70.1	2.2	43	58	–	–	76
	Radiation		56					–	–	84
Franco et al. (30)	Observation	–	7	67	2.7	38	63	–	–	–
	Radiation		7					–	–	–
	ChemoRT		2					–	–	–
Boyer et al. (6)	Observation	27.8	25	74	1.6	–	–	52	–	92
	Radiation		20					20	–	78
Clark et al. (8)	Observation	27.6	36	70	1.1	–	–	–	65	–
	Radiation		66					–	80	–
Pectaside et al. (35)	Observation	24	18	68	–	–	–	5	10	50
	Radiation		3					0	80	88
	ChemoRT		1					0	–	–
Jabbour et al. (24)	Observation	–	73	72	<2.0	37	63	–	–	38
	Radiation		36					–	–	62
	ChemoRT		9					–	–	–
Soult et al. (45)	Observation	26	13	71.3	2.0	11	88	15	20	–
	Radiation		13					23	38	–
Eich et al. (10)	Observation	22	14	73	–	42	58	86	20	–
	Radiation		16					62	38	–
Lok et al. (28)	Observation	51	0	74	–	64	35	–	–	–
	Radiation		40					10	–	70
Hui et al. (23)	Observation	26	11	79	<2.0	35	65	45	–	–
	Radiation		165					–	–	–
	ChemoRT		29					–	–	–
Allen et al. (3)	Observation	40	196	69	1.5	25	75	14	–	–
	Radiation		41					8	–	–
Poulsen et al. (42)	Observation	26	0	67.5	–	77	22	–	–	–
	Radiation		0					–	–	–
	ChemoRT		50					44	–	–
Wobser et al. (50)	Observation	–	–	80	–	40	60	–	–	–
	Radiation		1					–	–	–
	ChemoRT		4					60	0	–
Poulsen et al. (39)	Observation	22	0	67	<2.0	65	35	–	–	–
	Radiation		0					–	–	–
	ChemoRT		40					–	–	–
Poulsen et al. (37, 41)	Observation	56	17	76		–	–	–	10	–
	Radiation		29					–	40	–
	ChemoRT		6					–	–	–
Kim et al. (25)	Observation	–	269	77	<2.0	–	–	–	–	–
	Radiation		269					–	–	–
Lawenda et al. (26)	Observation	–	5	68.8	<2.0	33	67	60	–	–
	Radiation		3					0	–	–
	ChemoRT		1					100	–	–
Poulsen et al. (37)	Observation		0		–	55	45	–	–	–
	Radiation	41.5	62	72.5				42	–	–
	ChemoRT	29.5	40	67				25	–	–
Tai et al. (46)	Observation	25	21	77	<2.0	–	–	67	–	–
	Radiation		3					–	–	–
	ChemoRT		1					–	–	–
Fenig et al. (11, 14)	Observation	–	15	63	3.0	–	–	80	–	–
	Radiation		4					9	–	–
Eng et al. (14)	Observation	39.6	15	69	–	–	–	–	–	58
	Radiation		4					41	–	–
Boyle et al. (7)	Observation	36	17	68	–	–	–	71	–	–
	Radiation		10					–	–	–
Gillenwater et al. (18)	Observation	–	34	68.4	<2.0	–	–	97	–	–
	Radiation		26					50	–	–
Wong et al. (51)	Observation	–	16	80	<2.0	–	–	94	–	–
	Radiation		11					0	–	–
	ChemoRT		1					–	100	100

*Observation, surgery only treatment group; RT, received postoperative radiation therapy; ChemoRT, received postoperative chemoradiation therapy; F/U, follow up; LN, regional lymph node involvement; OS, overall survival; LC, local control*.

Adjuvant radiotherapy and chemoradiotherapy were both shown to be advantageous in LC compared with surgical excision alone. Three-year LC was 45.1% greater for the radiotherapy arm (64.6%) than the observation group (19.5%). Subset analysis demonstrated a 44.3% difference in 3-year LC for tumors <2 cm for the radiotherapy arm (75.9%) than the observation group (31.6%), and a 56.7% difference in 3-year LC for tumors ≧2 cm for the radiotherapy arm (64.7%) than the observation group (8%). These outcomes existed despite the fact that there were more patients with later stage disease in the radiation cohort. Chemoradiotherapy did not demonstrate any added benefits compared with radiotherapy alone in regards to LC.

Based on the pattern that current studies reveal, we recommend that a large prospective trial should be conducted to evaluate the true effect of postoperative radiation. Adjuvant radiotherapy was statistically significant for improvement of both OS and local recurrence. There was no statistically significant difference in any control or recurrence parameters assessed between postoperative RT and postoperative chemoRT arms. Adjuvant chemoRT did not significantly improve OS compared with radiotherapy alone. However, analysis of the primary tumor characteristics revealed that tumor stages and regional lymph node involvement were not equally represented among the three treatment cohorts. In particular, 56% of the patients receiving chemotherapy had stage II tumor and 10% of the patients had stage III tumor; both are significantly more common than patients receiving postoperative radiation therapy (39 and 0.5%, respectively). Also, 65% of the chemoRT patients were positive for clinical nodal involvement, compared to 35% in the overall cohort. In this review, chemoRT group had significantly fewer points of reference (*n* = 301) than the RT group (*n* = 1689). Chemotherapeutics may play a role in more advanced MCC, though limited data makes their role unproven. ChemoRT should also be considered in patients who are unresponsive to radiotherapy alone. Addition of chemotherapeutics should be used cautiously due to its toxicity. ChemoRT was shown to have statistically significant increase in both acute (*P* = 0.002) and chronic (*P* < 0.001) toxicity when compared with radiotherapy alone.

Merkel cell carcinoma is a cancer that has great metastasis potential. Though some physicians use size as a factor in determining the use of adjuvant treatments, no definitive guidelines have been published. In this review, we compared the mean primary tumor size among various publications. We separated all studies into one of two groups; Group 1 had a mean tumor size <2 cm and Group 2 had a mean tumor size ≧2 cm. This division has a major limitation; the groups are based on the mean of the study, not the mode. This division may skew the size of the majority of that study secondary to a few extremely large tumors. Though this classification system is not ideal, the results of this review support the use of radiotherapy regardless of tumor size. Mojika and colleagues (33) analyzed retrospective data from 1665 patient from the National Cancer Institute; this data suggested that overall median survival in months of patients were improved through the use of adjuvant radiation therapy in comparison to surgery alone, despite the size of the primary tumor. They further suggest that survival improvement was most prominent when primary tumors are larger than 2 cm.

Future research endeavors warrant comparison of radiotherapy and chemoradiotherapy adjuvants for advance stage MCC. This comparison will directly show if there is any added benefit to chemotherapeutics. Furthermore, it will also establish data from which recommendations for adjuvant treatments may be established based on clinical staging. Current study did not yield enough well documented cases to compare the two cohort directly. While this comprehensive retrospective analysis in a broad patient population reflects an advantage for postoperative radiation, there could be certain prognostic factors in a given patient that may favor surgery alone. Many variables may play a role in the success of treatment for MCC. Primary tumor size, nodal involvement and presence of metastasis should be recorded for points of data for future research. These variables may play a critical role in the establishment of treatment guidelines for MCC as well.

This review has several limitations; mostly a product of the retrospective nature of the studies included which is insufficient basis to recommend standard of care. Poulsen conducted the only three prospective studies conducted on postoperative management of MCC, and they all addressed adjuvant chemoradiation against adjuvant radiation alone (37, 39, 42). No randomized trial has ever been conducted regarding the role of radiation in MCC and the current clinical trials all address the use of certain immunotherapies that have been successful in other skin cancers. Although we attempted to make cohorts similar through certain subset analyses, a true comparison of outcomes of patients with the same stage, tumor size, or nodal involvement could not be accomplished due to a lack of original patient data. The trend identified by current study support the use of postoperative radiation for MCC. The data on postoperative chemoradiation, on the other hand, was restricted enough by the studies limitations so that no trend could be discovered.

## Conclusion

This systematic review demonstrates that the use of adjuvant radiotherapy in the treatment of MCC, regardless of tumor size, location, or nodal involvement, resulted in survival and control benefit reported in the literature. However, standard documentation of retrospective data and large scale prospective studies are needed to consolidate current trend. The use of adjuvant chemoradiotherapy remains unproven, although there may be a benefit in systemic disease. Establishment of treatment guidelines is needed.

## Conflict of Interest Statement

The authors declare that the research was conducted in the absence of any commercial or financial relationships that could be construed as a potential conflict of interest.
